# IL-17A plays a critical role in RSV infection in children and mice

**DOI:** 10.1186/s12985-023-01990-8

**Published:** 2023-02-15

**Authors:** Xin Long, Jun Xie, Luo Ren, Guangyuan Yu, Enmei Liu, Yu Deng, Xiaoru Long

**Affiliations:** 1 Department of Respiratory Medicine, Children’s Hospital of Chongqing Medical University, National Clinical Research Center for Child Health and Disorders, Ministry of Education Key Laboratory of Child Development and Disorders, No.136, Zhongshan 2nd Road, Yuzhong District, Chongqing, 400014 People’s Republic of China; 2 Department of Infection, Children’s Hospital of Chongqing Medical University, National Clinical Research Center for Child Health and Disorders, Ministry of Education Key Laboratory of Child Development and Disorders, Chongqing, 400014 People’s Republic of China; 3 Chongqing Key Laboratory of Child Infection and Immunity, Chongqing, 400014 People’s Republic of China

**Keywords:** Respiratory syncytial virus, IL-17A, Airway inflammation

## Abstract

**Background:**

IL-17A is a pleiotropic cytokine and intimately associated with asthma, but its role in respiratory syncytial virus (RSV) infection is conflicting in the literature.

**Methods:**

Children hospitalized in the respiratory department with RSV infection during RSV pandemic season of 2018–2020 were included. Nasopharyngeal aspirates were collected for pathogen and cytokines determination. In the murine model, RSV intranasal administrations were performed in wild-type and IL-17A-/- mice. Leukocytes and cytokines in bronchoalveolar lavage fluid (BALF), lung histopathology, and airway hyperresponsiveness (AHR) were measured. RORγt mRNA and IL-23R mRNA were semi-quantified by qPCR.

**Results:**

IL-17A increased significantly in RSV-infected children and was positively associated with pneumonia severity. In the murine model, IL-17A significantly increased in BALF of mice with RSV infection. Airway inflammation, lung tissue damage and AHR were significantly alleviated in wild-type mice following IL-17A neutralization and in the IL-17A-/- mice. IL-17A decreased by removing CD4^+^ T cells but increased by depleting CD8^+^ T cells. IL-6, IL-21, RORγt mRNA and IL-23R mRNA dramatically increased in parallel with the rise of IL-17A.

**Conclusions:**

IL-17A contributes to the airway dysfunctions induced by RSV in children and murine. CD3^+^CD4^+^T cells are its major cellular sources and the IL-6/IL-21-IL-23R-RORγt signaling pathway might participate in its regulation.

## Background

RSV is the major cause of viral bronchiolitis in infants, with attack rates approaching 100% by the third RSV season, and triggers wheezing disease in later childhood [1]. Disease burden is even heavier in low- and middle-income countries, and the occurrence and severity of infection remains highly unpredictable [2]. However, despite more than 50 years of research, a safe and effective vaccine remains elusive and treatment remains supportive [3]. Till now, the underlying mechanisms in the setting of RSV infection are as-yet contradictory.

The involvement of IL-17A in the pathophysiology of airway disorders is of great interest [4, 5]. It is important in several diseases, including chronic obstructive pulmonary disease, cystic fibrosis, and asthma, and clinical trials have been planned to target IL-17A as a treatment for patients with inadequately controlled or steroid-resistant asthma [6]. But its role in RSV infection has not been understood completely, and previous studies have come to contradictory conclusions. Faber and colleagues [7] reported that IL-17A levels were higher in bronchoalveolar lavage fluid (BALF) from pediatric patients (13 months and below) with non-ventilated RSV disease at admission and at discharge compared with BALF from more severe, ventilated cases. While other researchers contrarily noticed that IL-17A accounted for RSV-induced mucus secretion [8].

IL-17A can be produced by Th17 cells and many innate immune cells such as dendritic cells, iNKT cells etc [9]. Wang et al. [10] have identified a population of memory/effector IL-17^+^ Th2 cells, which were significantly increased in the peripheral blood of patients with atopic asthma. These cells can concomitantly produce IL-17A and type 2 cytokines, and have induced more severe asthma than classical Th2 and Th17 cells. Another study has proved that ILC2_17s_ cells, a population of innate lymphoid cells (ILCs), are the major source of IL-17A [11]. TGF-β, IL-6, and IL-23 have been implicated in the differentiation of Th17 cells, among which, IL-23, in particular, has been shown to be a potent inducer of IL-17A not only in CD4 T cells but in CD8 T cells, γδ T cells, NK cells and neutrophils [12]. Thus, the molecular regulation of IL-17A production is complex. And it is important to investigate cellular sources for IL-17A in the lung and determine molecular regulation of IL-17A production during RSV infection.

In the present study, we aimed to further elaborate on the role of IL-17A in the condition of RSV infection by including RSV-infected children hospitalized in the respiratory department and using IL-17A knock-out mice and neutralizing antibody. It was found that IL-17A contributed to airway inflammation and AHR after RSV infection. CD3^+^CD4^+^ T cells promote but CD3^+^CD8^+^ T cells suppress IL-17A production. The IL-6 /IL-21-IL-23R-RORγt signaling pathway was elevated in parallel with IL-17A. Our findings highlight that IL-17A plays a critical role in the progression of RSV infection.

## Methods

### Study population

Children were enrolled in this study who were admitted to the Department of Respiratory Medicine, Children’s Hospital, Chongqing Medical University during RSV pandemic season (November to February next year) from 2018 to 2020 and diagnosed as respiratory infection. Immediately following hospital admission, nasopharyngeal aspirates (NPAs) were prospectively collected from all subjects, and venous blood was taken for routine biologic tests. Signs and symptoms were recorded on a standard form used in our hospital. Severe pneumonia was defined in accordance with the guideline of WHO [13].

In addition, the control group was those hospitalized in the surgery department who had undergone surgery without any sign of respiratory infection.

NPAs were collected from those patients mentioned above and tested for multiple virus and bacteria.

This study was approved by the Ethics Committee of the Children’s Hospital of Chongqing Medical University (number: (2015) NO. (77-1)).

#### Virus detection and cytokine measurement

After NPA was collected, it was gently aspirated with a Pasteur pipette and then expelled repeatedly until mixed uniformly: 0.2 mL of the specimen was then transferred to another tube for cellular identification and cytokine estimation. To this sample, about four times its volume of 0.1% dithiothreitol was added, and the whole was mixed with a Pasteur pipette, vortexed for 15 s, and rocked on a bench rocker for 15 min. The suspension was subsequently centrifuged at 1500 r.p.m. for 10 min. The cell-free supernatant was stored at − 80 °C.

The rest of the original NPAs with tested for viruses. Virus nucleic acid extraction (QIAampMinElute Virus Spin Kit, Qiagen, Hilden, Germany), cDNA synthesis (SuperScript III First-Strand Synthesis System, Invitrogen, California, USA) as well as Multiplex nested polymerase chain reaction (PCR) were described previously [14]. Every NPA was tested for viruses: RSV subtypes A and B (RSV_A, RSV_B); influenza virus (IFV) subtypes A, B and C (IFVA, IFVB, IFVC); human coronaviruses (HCoV); metapneumovirus (MPV); parainfluenza virus type 1 to 4 (PIV1-4); adenovirus (ADV); human bocavirus type 1 (HBoV1) and human rhinovirus (HRV) subtypes A and C (HRVA, HRVC) [14]. In addition, NPAs samples were determined for bacteria as previously described [15]. Only specimens positive for RSV_A alone were included in this study.

Cytokines of IL-17 were measured using Human Premixed Multi-Analyte Kit (R&D, USA), and then analyzed according to the manufacturer's instructions (Wayen Biotechnologies. Inc., China) using Luminex 200 system (Luminex Corporation, USA). Bio-Plex Manager software was used to calculate cytokine concentrations on the basis of fluorescence values derived from a recombinant cytokine standard included in the 96-well plate. A nonlinear least-squares minimization algorithm generated a curve fitted by a five-parameter logistic equation and determined the high and low limits of detection.

### Mice

Female wild-type C57BL/6 mice and BALB/c mice at 6 weeks were purchased from the Chongqing Medical University Animal Laboratory. IL-17A-/- mice on a C57BL/6 background were a kind gift from Professor Bin Li of the Third Military Medical University in Chongqing, China. The mice were bred under accredited specific pathogen-free conditions in separate filter-top cages according to the experimental design. All experiments involving animals were in accordance with the Guide for the Care and Use of Laboratory Animals and approved by the Institutional Animal Care and Committee (IACUC), which is accredited by the Association for Assessment and Accreditation of Laboratory Animal Care International, China, and Experimental Animal Committee of the Chongqing Medical University (license numbers: SCXK (Yu) 2012–0001 and SYXK (Yu) 2012–0001).

### Virus stocks and mice inoculation

Human RSV (strain A2) was obtained from American Type Culture Collection, and the viral titer was determined by plaque assay. Mice were held upright after sedation and infected intranasally with 7.5 × 10^7^ PFU RSV in a total volume of 100 μl (RSV group), or sham-infected with 100 μl UV-inactivated RSV (control group) on day 0.

### Inflammatory cell counts

BALF was collected as previously described [16]*.* Briefly, mice were anesthetized and tracheae were cannulated with ice-cold PBS. BALF was centrifuged. The supernatants were collected and stored at − 80 °C for cytokines detection. The cell pellet was re-suspended with 1 ml PBS, and the total cells were quantified by automated cell counter (Count Star, China).

### Lung histology

For histology studies, left-lung lobes from mice were removed, fixed in 10% formalin, cut into 5 μm sections, and stained with hematoxylin and eosin solution (Sigma-Aldrich, Sigma MHS-16 and Sigma HT110-1-32, respectively, St. Louis, MO, USA). The degree of airway inflammatory cell infiltration was scored as previously described [17].

### Airway hyperresponsiveness (AHR)

AHR was evaluated by measuring the respiratory system resistance (Rrs) with an invasive lung function test as previously described [18]. Briefly, mice were anesthetized with pentobarbital sodium (Sigma, 90–100 mg/kg, intraperitoneally) and connected via a tracheostomy tube to a computer-controlled small animal ventilator (flexiVent; Scireq, Montreal, Canada) at 150 breaths/min with a tidal volume of 10 mL/kg. Subsequently, mice were exposed to aerosolized acetyl-b-methylcholine (Sigma-Aldrich, Saint Louis, MO, USA) at increasing doses: 0, 3.125, 6.25, 12.5, 25, and 50 mg/ml. At each dose, the Rrs was recorded and the average Rrs values were calculated.

### Cytokine analysis

The concentrations of IL-17A, IL-6, IL-21, IFN-γ, IL-18 and IL-23 in BALF of mice were measured using mouse-specific ELISA kits, according to the manufacturer´s instructions (eBioscience, CA, USA).

### Flow cytometry analysis

Single-cell suspensions of the lung were prepared and cells were incubated with PMA (50 ng/ml; Sigma), ionomycin (1000 ng/ml; Sigma), and GolgiPlug-containing brefeldin A (BD Biosciences) for 4–6 h as described previously [17]*.* The cells were harvested and blocked with antibody to mouse CD16/CD32 (Fc Block, BD). Samples were immunostained with antibody to mouse CD3, CD4, CD8, or isotype control conjugated with PerCP-cy5.5, FITC or PeCy7 for 30 min on ice and then fixed with 1% Formaldehyde in FACS Staining Buffer. The indicated antibodies were obtained from eBioscience (San Diego, CA). For intracellular IL-17A detection, cells were fixed and permeabilized with CytoFix/CytoPerm solution (554722; BD) and Perm/Wash buffer (554723; BD) and then stained with APC-conjugated anti-IL-17 mAb (BD Biosciences). Stained samples were measured on a flow cytometer, FACSCalibur (BD Biosciences). The data were analyzed using CellQuest software (BD Biosciences).

### Gene expression assessment

Total RNA was isolated via extraction from whole lung homogenates using TRIzol (Invitrogen, CA, USA). The PrimeScript RT reagent kit (TaKaRa, Japan) was then used to prepare cDNA from this isolated RNA. Power SYBR® Green PCR Master Mix (Life Technologies) was next used to perform qPCR with the following target gene primer sets being used: RORγt-F, 5′-TGCCAACAACCACACAGTCT-3′, RORγt-R, 5′-AGGACGGTTGGCATTGATGA-3′; IL-23R-F, 5′- GGTCCAAGCTGTCAATTCCCTAGGC-3′, IL-23R-R, 5′- AGCCCTGGAAATGATGGACGCA-3′; β-actin-F, 5′-TGGCATTGTTACCAACTGGGAC-3′, β-actin-R, 5′-TCACGGTTGGCCTTAGGGTTC-3′.

### Cell depletion

To assess the effects of CD3^+^CD4^+^ T cells and CD3^+^CD8^+^ T cells, BALB/c mice were intraperitoneally injected with 100 μg rat anti-mouse CD4 (clone YTS177, Bioxcell) or CD8a antibodies (clone 116-13.1, Bioxcell) respectively on days 14, 17 and 20 post RSV infection. Control antibodies were administered similarly and disease parameters were assessed on day 21.

### Antibodies treatment

To neutralize IL-17A, mice were injected with 100 μg anti-IL-17 antibody (clone 50104, R&D, Abingdon, UK) intraperitoneally on days 14, 16, 18, and 20 after RSV challenge. The corresponding isotype-matched controls (R&D Systems, Minnesota, USA) were given similarly. Disease parameters were assessed on day 21.

### Statistical analysis

Statistical analyses were performed with SPSS (version 22.0), Graphpad Prism (version 7.0), medcalc (version 11.4). Data related to clinical samples were analyzed by Wilcoxon rank-sum test. The correlation between the length of hospitalized days and levels of cytokines were measured by Spearman’s rank correlation test. The discrimination of IL-17A for risk of severe pneumonia was determined using the area under the receiver operating characteristic (ROC) curve. *P* values were judged significant if they were less than 0.5.

The results from murine models are expressed as means ± SEM. Statistical significance was assessed by one- or two-way analysis of variance (ANOVA). One-way ANOVA with repeated measures was used to analyze the significant differences among the three groups. Two-way ANOVA was used to analyze the significant differences between two variables, especially for Rrs values. If an overall test was significant, Tukey’s test was utilized for specific comparisons between individual groups. Differences were considered to be significant at *P* < 0.05.

## Results

### IL-17A was correlated to the severity of pneumonia in children hospitalized with RSV infection

46 cases infected with RSV_A alone and 25 control cases were included in the present study. Details of RSV-infected children were shown in Table 1.

**Table 1:** Demographic and clinical characteristics of RSV-infected children

	RSV-infected N = 46 (%)
Age (months)^a^	4 (1–51)
Male	28 (60.9)
Symptoms	
Wheezing	31 (67.4)
SpO2 < 90%	5 (10.9)
Dyspnea	8 (17.4)
Duration (days)^a^	12 (7–68)
Length of stay (days)^a^	7 (4–27)
Severe pneumonia	25 (54.3)

^a^Data were shown with median and range

**Fig. 1:**
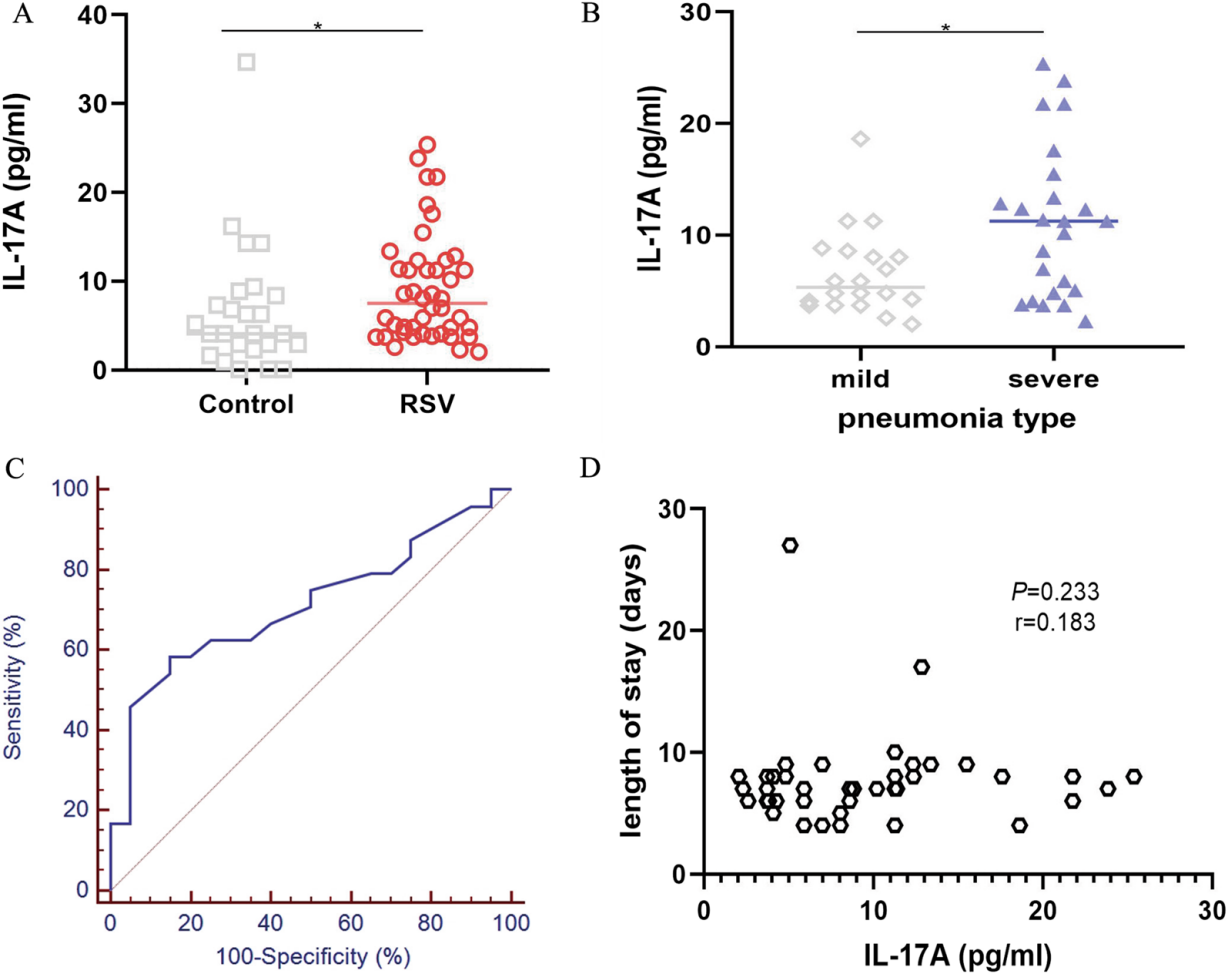
The association between levels of IL-17A and RSV infection**.** **A** IL-17A in RSV-infected group was higher than those in the control group. **B** IL-17A was significantly increased in children with severe pneumonia as compared to the mild group among RSV infection group. **C** IL-17A showed the limited power of discrimination for severe pneumonia in the RSV-infected group. **D** no significant correlation was found between the length of hospitalized days and IL-17A. *P < 0.05, **P < 0.01, ***P < 0.001

IL-17A was higher in RSV infection group than those in the control group (*P* < 0.05, Fig. 1A). Among RSV infection group, IL-17A was positively related to the severity of pneumonia (*P* < 0.05, Fig. 1B). In addition, IL-17A showed limited predictive power for the risk of severe pneumonia (*P* < 0.05, AUC:0.712 with 95% CI:0.556–0.839; Fig. 1C). Unexpectedly, IL-17A was not correlated with the length of hospitalized days. (*P* > 0.05, Fig. 1D).

**Fig. 2:**
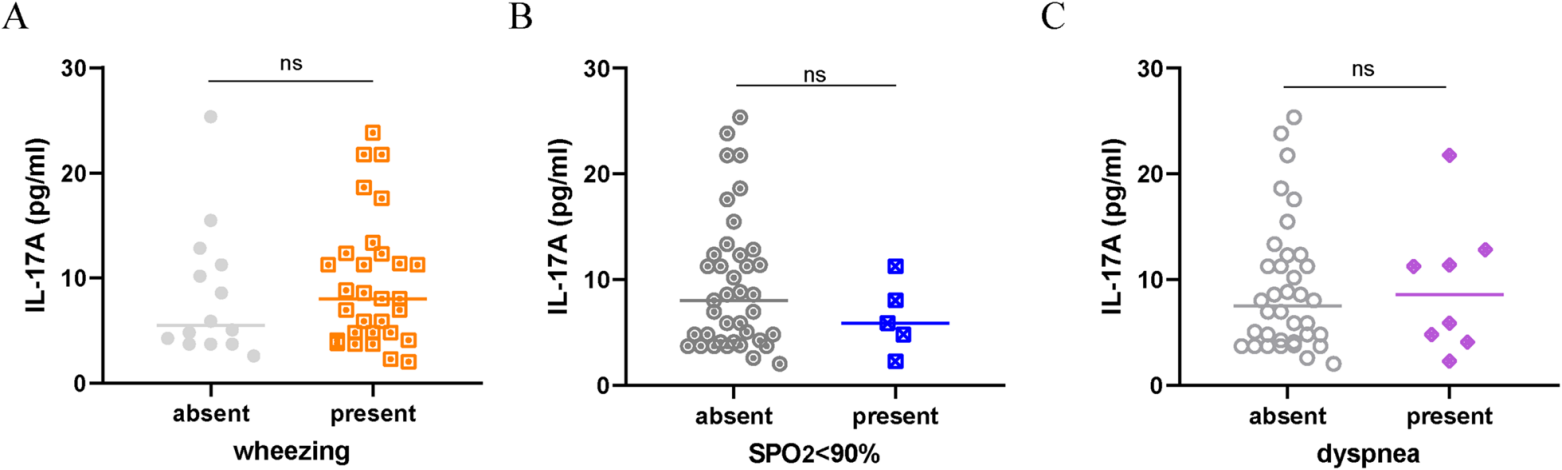
IL-17A correlated with symptoms in the RSV-infected group. **A**–**C**: no significant difference in IL-17A was found between groups absent or present with wheezing, SpO_2_ < 90%, or dyspnea, respectively. *P < 0.05, **P < 0.01, ***P < 0.001

Then the relationship between the presence of symptoms and levels of IL-17A was tested. Surprisingly, IL-17A was not correlated to any symptoms (wheezing, presence of SpO_2_ < 90%, or dyspnea, Fig. 2A–C).

### IL-17A contributes to persistent airway disorders in mice with RSV infection

**Fig. 3:**
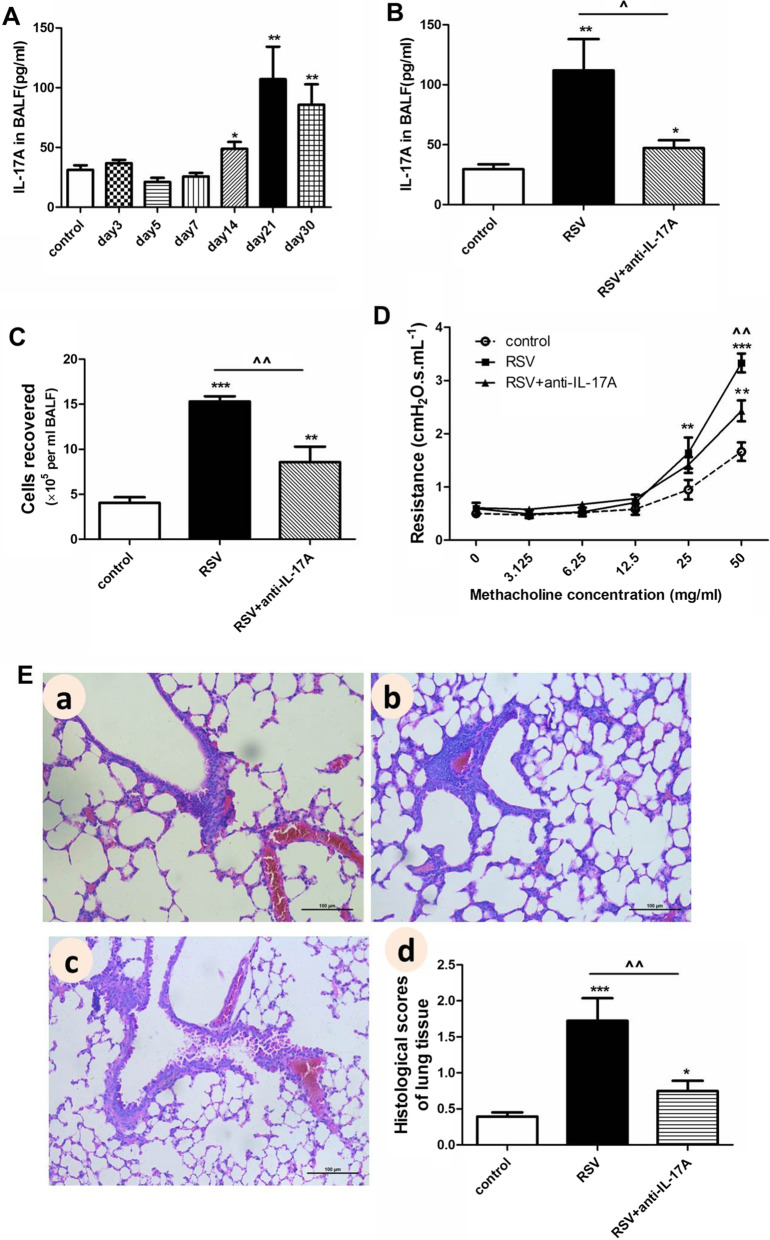
Neutralization of IL-17A alleviates the persistent airway pathophysiology during RSV infection. Mice were treated with neutralizing anti-IL-17A or isotype control antibody on days 14, 16, 18, and 20 post RSV infection. Disease parameters were assessed on day 21. **A–B**: Levels of IL-17A in BALF. **C**: Total inflammatory cells isolated from BALF. **D**: AHR in response to increasing doses of methacholine. **E**: Representative HE staining and histological scores (HPS) of lung tissues (original Magnification × 200). a: control group (sham-infected and PBS treated mice); b: RSV group (RSV-infected and isotype control antibody-treated mice); c: RSV + anti-IL-17A group (RSV-infected and neutralizing anti-IL-17A antibody-treated mice); d: HPS. Graphs are represented as the mean±sem. Data are representative of two independent experiments performed on 6 animals per group. *P < 0.05, **P < 0.01, ***P < 0.001 shown comparing the control group with the other groups; ^*P* < 0.05, ^^*P* < 0.01, shown comparing the RSV group with the RSV + anti-IL-17A group

**Fig. 4:**
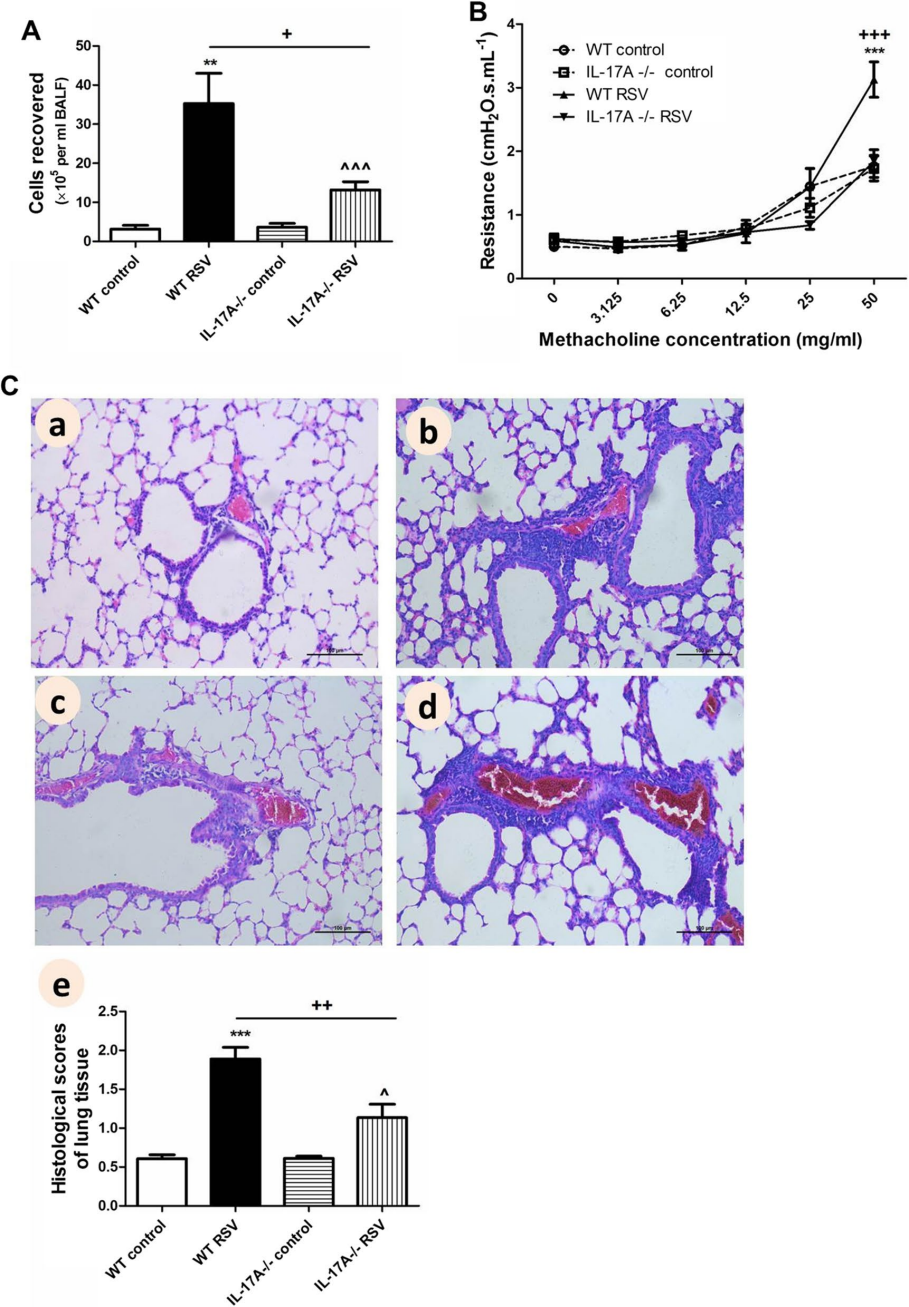
RSV-associated airway inflammation and AHR were decreased in IL-17A-/- mice. Wild-type C57BL/6 mice and IL-17A-/- mice (on C57BL/6 background) were sacrificed on day 21 after inoculation with RSV. **A** Total inflammatory cells isolated from BALF. **B** AHR in response to increasing doses of methacholine. **C** Representative HE staining and HPS of lung tissue sections (original Magnification × 200). a: control wild type mice; b: RSV-infected wild type mice; c: control IL-17A-/- mice; d: RSV-infected IL-17A-/- mice; e: HPS. Graphs are represented as the mean±sem. Data are representative of two independent experiments performed on 6 animals per group. **P < 0.01, ***P < 0.001 shown comparing the control group with the RSV group in wild type mice; ^*P* < 0.05, ^^^*P* < 0.001 shown comparing the control group with the RSV group in IL-17A-/- mice;^ + ^*P* < 0.05, ^+ +^*P* < 0.01, ^+++^*P* < 0.001 shown comparing the wild type mice with the IL-17A-/- mice

To evaluate the role of IL-17A in RSV infection, we first detected IL-17A expression in the murine model. BALF samples were collected on days 3, 5, 7, 14, 21, and 30 post RSV infection. As shown in Fig. 3A, IL-17A was significantly increased on days 14 (*P* < 0.05), 21 (*P* < 0.01), and 30 (*P* < 0.01) as compared to the control mice group. Wild type BALB/c mice were next treated with neutralizing anti-IL-17A antibody (RSV + anti-IL-17A). As shown in Fig. 3B, IL-17A levels markedly reduced in the RSV + anti-IL-17A group as compared to the RSV group (*P* < 0.05). Meanwhile, inflammatory cells in BALF (Fig. 3C , *P*< 0.01), AHR (Fig. 3D, *P *< 0.01, at methacholine concentrations of 50.0 mg/ml), lung tissue damage (Fig. 3E a-c) and histological scores (HPS, Fig. 3E d , *P*< 0.01) all dramatically alleviated in the RSV + anti-IL-17A group as compared to the RSV group. To further verify the pathogenic role of IL-17A, we infected IL-17A knock-out mice (IL-17A-/-, on C57BL/6 background) with RSV (IL-17A-/- RSV). As shown in Fig. 4A, inflammatory cells counts in BALF in IL-17A-/- RSV group were higher as compared to the control IL-17A-/- mice group (IL-17A-/- control, *P* < 0.001); but were much lower as compared to the RSV-infected wild-type C57BL/6 mice group (WT RSV, *P* < 0.05). AHR kept similar between the IL-17A-/- RSV group and the IL-17A-/- control group in response to the increasing concentrations of methacholine challenge (Fig. 4B). AHR at methacholine concentrations of 50.0 mg/ml was markedly higher in the WT RSV group as compared to the IL-17A-/- RSV group (*P* < 0.001). In addition, worse lung tissue damage (Fig. 4C a–d) and higher HPS (Fig. 4C e) were found in IL-17A-/- RSV group as compared to the IL-17A-/- control group (*P* < 0.05); but these parameters in IL-17A-/- RSV group were much lighter as compared to the WT RSV group (*P* < 0.01). Taking together, these results indicated that IL-17A contributed to the persistent airway inflammation and AHR during RSV infection.

### *CD3* + *CD4* + *T cells promote but CD3* + *CD8* + *T cells inhibit IL-17A production*

**Fig. 5:**
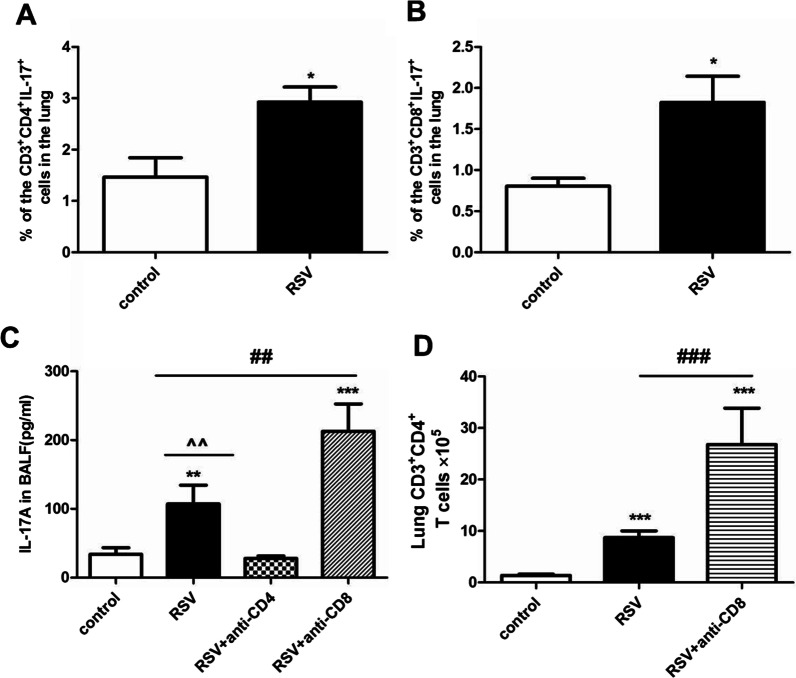
CD3^+^CD4^+^ T cells promoted but CD3^+^CD8^+^ T cells inhibited IL-17A over-production during RSV infection. CD3^+^CD4^+^IL-17^**+**^ T cells (**A**) and CD3^+^CD8^+^ IL-17^**+**^ T cells (**B**) in the lung tissue were analyzed with flow cytometry on day 21 post RSV challenge. To further identify the sources of IL-17A, mice were treated with anti-CD4 or anti-CD8a antibodies on days 14, 17, and 20. IL-17A levels were assessed on day 21 (**C**). CD3^+^CD4^+^ T cells were detected after the depletion of CD3^+^CD8^+^ T cells (**D**). Graphs are represented as the mean±sem. Data are representative of two independent experiments performed on 6 animals per group. *P < 0.05, **P < 0.01, ***P < 0.001 shown comparing the control group with the RSV group; ^^*P* < 0.01 shown comparing the RSV group with the RSV + anti-CD4 group; ^##^*P* < 0.01, ^###^*P* < 0.001 shown comparing the RSV group with the RSV + anti-CD8 group

We next identified the cellular sources of IL-17A induced by RSV. As shown in Fig. 5A, B, percentages of both CD3^+^CD4^+^IL-17^+^ T cells (*P* < 0.05) and CD3^+^CD8^+^ IL-17^+^ T cells (*P* < 0.05) significantly increased on days 21 post RSV infection. Subsequently, we depleted these two cells using specific antibodies. As shown in Fig. 5C, IL-17A levels in BALF of RSV infected group administrated by anti-CD4 antibody dramatically reduced equally to the control group (*P* < 0.01 vs. the RSV group; *P* > 0.05 vs. the control group). However, IL-17A levels unexpectedly markedly increased following anti-CD8 antibody treatment as compared to the control group (*P* < 0.001) and the RSV group, respectively (*P* < 0.01). Thus, it’s plausible to propose that CD3^+^CD4^+^ T cells promoted while CD3^+^CD8^+^ T cells inhibited IL-17A production. We further evaluated the effects of the depletion of CD3^+^CD8^+^ T cells on CD3^+^CD4^+^ T cells. As shown in Fig. 5D, following anti-CD8 antibody administration, CD3^+^CD4^+^ T cells were further substantially boosted in the RSV + anti-CD8 group as compared to both the control group (*P* < 0.001) and the RSV group (*P* < 0.001). Taking together, these results indicated that CD3^+^CD4^+^ T cells were important sources of IL-17A induced by RSV infection; and CD3^+^CD8^+^ T cells seemed to protect against RSV-associated disorders by inhibiting CD3^+^CD4^+^ T cells and IL-17A production.

### IL-6 / IL-21-IL-23R-RORγt signaling pathway is activated in parallel with the increase of IL-17A

**Fig. 6:**
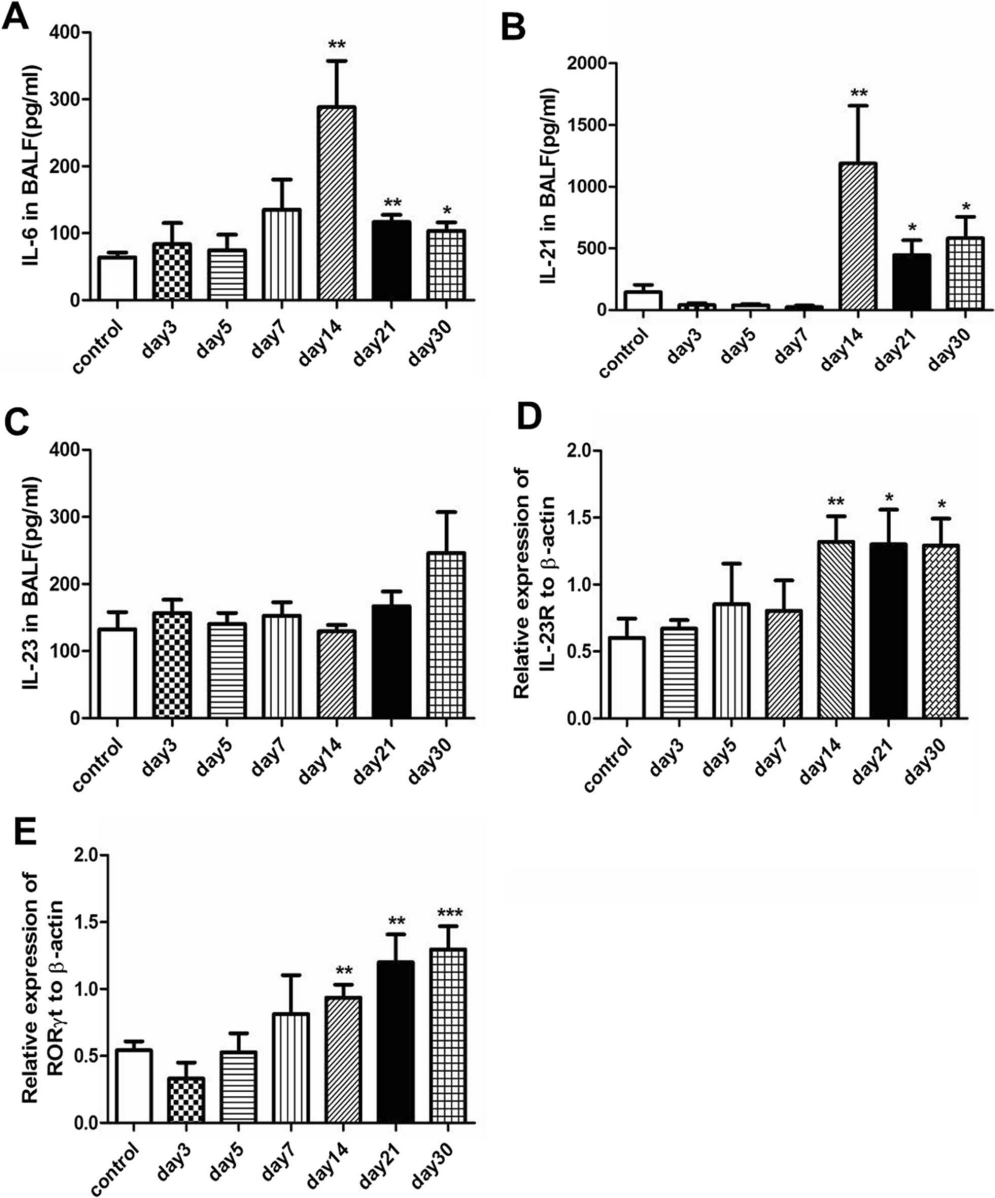
IL-6 / IL-21 -IL-23R- RORγt signaling pathway is elevated during RSV infection. IL-6 / IL-21 -IL-23R- RORγt signaling pathway is involved in regulating IL-17A in other models [12]. We thus detected this pathway in our study. **A** IL-6 levels in BALF. **B** IL-21 levels in BALF. **C** IL-23 levels in BALF. **D** Gene expression of IL-23R. **E** Gene expression of the transcription factor RORγt. Graphs are represented as the mean±s.e.m. Data are representative of two independent experiments performed on 6 animals per group. *P < 0.05, **P < 0.01, ***P < 0.001 shown comparing the RSV group with the control group

IL-23 is known as a critical regulator of IL-17A production. And IL-6 can induce IL-21, which can subsequently up-regulate IL-23R, eventually facilitating expression of RORγt (the master transcription factor for IL-17A) [12]. We then assessed this signaling pathway in the current model. IL-6 (Fig. 6A) and IL-21 (Fig. 6B) significantly increased on days 14, 21 and 30, respectively (*P* < 0.01, 0.01, 0.05 for IL-6; *P* < 0.01, 0.05, 0.05 for IL-21) as compared to the control mice group. Although IL-23 levels (Fig. 6C) showed no obvious changes after RSV infection, IL-23R mRNA (Fig. 6D) strikingly elevated on days 14 (*P* < 0.01), 21 (*P* < 0.05) and 30 (*P* < 0.05), respectively. Moreover, we also observed that RORγt mRNA (Fig. 6E) consistently increased on days 14 (*P* < 0.01), 21 (*P* < 0.01) and 30 (*P* < 0.001), respectively. Taking together, these results indicated that the IL-6 / IL-21 -IL-23R- RORγt signaling pathway might participate in regulating IL-17A production during RSV infection.

## Discussion

In the current study, we have shown that IL-17A was positively correlated with the severity of pneumonia in children and contributed to airway dysfunctions in a murine model with RSV infection. CD3^+^CD4^+^ T cells are its major cellular sources. And IL-6 / IL-21-IL-23R- RORγt signaling pathway might participate in regulating its production.

IL-17A has been known to play a critical role in the pathogenesis and progression of pulmonary inflammation caused by viruses. Recent reviews showed evidence that increased Th17 cell activation and IL-17 signaling were found in the plasma and lung of COVID-19 patients and were also linked to critical illness in COVID-19, which may lead to an increased likelihood of lung injury and respiratory failure [19, 20]. Maximillian et al. [21] suggested that airway neutrophilic inflammation activated by IL-17A in healthy volunteers was associated with subsequent symptomatic RSV infection. Studies on RSV-infected infants showed that IL-17A concentration in tracheal aspirates and BALF was higher in RSV-infected infants compared with the control group [22, 23], especially in those critically ill ones [24]. Furthermore, increased IL-17A facilitated neutrophil recruitment and led to pathologic consequences for infant lungs [22]. Thus, it can be concluded that IL-17A concentration in airway was associated with severe respiratory tract infection. Consistent with previous studies, our study demonstrated that IL-17A in NPAs were significantly higher in RSV-infected children than in the control group and were positively correlated to the severity of pneumonia related to RSV infection. IL-17A is a common pathogenic molecule regulating disease severity induced by respiratory viruses [25]. Several studies on mouse models had elaborated the mechanism of IL-17A contributing to lung damage: 1) mucus hyper-secretion in the airways, 2) alteration of effector CD8 T cell responses and 3) recruitment of neutrophils [8, 26]. Increased IL-17A level in mice BALF was correlated with the alterations in lung function and the persistent inflammation caused by influenza virus [25]. As for mice infected with RSV, anti-IL-17 treatment could alleviate the severity of immunopathology [25]. Another study showed that IL-17A tested in BALF was associated with pulmonary pathogenesis during RSV infection and exacerbation of allergic disease in a mouse model [24]. Our murine model indicated that IL-17A was significantly increased in RSV-infected mice during 14 to 21 post RSV infection and further neutralization of IL-17A in wild-type mice ameliorated the RSV-induced lung inflammation and disease parameters were markedly lower in IL-17A-/- mice. By using human specimens and mouse model, we confirm the detrimental role of IL-17A in the disease caused by RSV. However, we failed to find the association between clinical manifestation (hypoxia, dyspnea, and wheezing) and IL-17A levels in NPAs. Our mouse model indicated that IL-17A contributed to the airway hyperresponsiveness during late stage of RSV infection, while NPAs were collected at a single point in time, the failure to detect a difference may be due to the kinetics of IL-17A increases.

We subsequently investigated the cellular sources of IL-17A. Flow cytometry analysis revealed that CD3^+^CD4^+^IL-17^+^ T cells were strikingly increased on day 21 post RSV infection, and IL-17A consistently reduced equal to the control levels following anti-CD4 antibody treatment. Intriguingly, CD3^+^CD8^+^IL-17^+^ T cells count also dramatically increased on day 21, but IL-17A was unexpectedly vigorously boosted by anti-CD8 treatment. Furthermore, CD3^+^CD4^+^ T cells markedly augmented flowing the depletion of CD3^+^CD8^+^ T cells. Thus, it’s plausible to propose that CD3^+^CD8^+^ T cells may exert protective effects against RSV challenge by suppressing IL-17A production. The CD8^+^ T cells responses have multiple roles including clearance of the viral infection and establishment of a type I immune environment. Joost et al. [27] described that transfer of CD8^+^ T cells from RSV-infected mice significantly ameliorated the airway hyperreactivity after the cockroach allergen (CRA) challenge. In addition, the transfer of CD8^+^ T cells substantially inhibited CRA-induced IL-5 and IL-13 production by lung lymph node cells, but significantly increased IFN-γ production. Influenza-specific effector memory CD8^+^ T cells could also inhibit allergic responses by antigen-independent production of IFN-γ [28]. CD8^+^ T cells are critical regulators of Th2-driven eosinophilic lung disease [29]*.* CD8^+^ T cell was reported to be increased in RSV severe infection group accompanied by the decrease of IL-17A compared to the moderate infection group [30]. While the mechanism of CD8^+^ T cells inhibiting IL-17A production in the condition of RSV infection needs to be further defined.

IL-23 is required for the accumulation and function of Th17 cells in mice infected with pathogenic microorganisms such as Citrobacter rodentii and in mice immunized with antigen in complete Freund’s adjuvant [31]. And IL-6 can induce IL-21, which subsequently up-regulates the expression of IL-23R. IL-6, IL-21, and IL-23 can each independently induce RORγt mRNA in activated CD4^+^ T cells. RORγt is necessary and sufficient in directing IL-17A transcription [12]. Our current results showed that IL-6, IL-21, IL-23R mRNA, RORγt mRNA all significantly increased simultaneously. But whether IL-17A production was regulated by the IL-6 / IL-21-IL-23R- RORγt signaling pathway remained to be decided.

## Conclusions

In summary, we confirm the contribution of IL-17A to the airway disorders induced by RSV and add new insights into the expanding research that tries to elucidate the complicated cellular sources and regulators of IL-17A. These results further highlight the potential to target IL-17A to treat RSV-associated recurrent wheezing and pneumonia.

## Data Availability

The datasets used during the current study are available from the corresponding author on reasonable request.

## Abbreviations

ADV: Adenovirus
AHR: Airway hyperresponsiveness
BALF: Bronchoalveolar lavage fluid
CRA: Cockroach allergen
HBoV: Human bocavirus
HCoV: Human coronaviruses
HPS: Histological scores
HRV: Human rhinovirus
IACUC: Institutional Animal Care and Committee
IFV: Influenza virus
ILCs: Lymphoid cells
MPV: Metapneumovirus
NPAs: Nasopharyngeal aspirates
PCR: Polymerase chain reaction
PIV: Parainfluenza virus
RSV: Respiratory syncytial virus
